# The Mechanisms of Sleep Function and Regulation for Health and Cognitive Performance

**DOI:** 10.3390/brainsci13121680

**Published:** 2023-12-06

**Authors:** Marco Fabbri

**Affiliations:** Department of Psychology, University of Campania Luigi Vanvitelli, 81100 Casert, Italy; marco.fabbri@unicampania.it; Tel.: +39-0823-275333

Although we spend about one third of our life sleeping, the function and regulation of sleep remain scientific enigmas. It has been widely proposed that sleep function and sleep regulation are inseparable, although there is a lack of consensus concerning their mechanisms. This Special Issue includes ten papers that highlight this topic from different points of view. For example, Barbato [1] presents a comprehensive review of the rapid eye movement (REM) density mechanisms as an index of sleep satiety and a sensitive measure of sleep homeostasis, in addition to the analysis of slow wave activity. The review clearly presents the relevance of REM density as a possible index of the intensity of REM sleep, which is generally altered in depressed patients. Given that the discovery of REM sleep has consistently influenced the scientific community in studying the sleep architecture and its association with cognitive function and psychophysical well-being, Barbato [1] presents an interesting analysis of how REM density can be considered a physiological marker of the “time to wake” during sleep.

Another perspective of the focus of the Special Issue is that of assessing patients with sleep disorders in their cognitive functioning. Fabbri et al. [2] categorized individuals from the general population into normal, subclinical, and moderate/severe sleep groups according to their Insomnia Severity Index (ISI) scores and requested participants to perform a visual dot-probe task. The results seemed to support the evidence for several cognitive models of insomnia, with a potential utility for the clinical assessment of insomnia. In a similar way, Castelnovo et al. [3] present the neuropsychological and neurophysiological assessment of a group of chronic insomniacs and long-term, high-dosage benzodiazepine (BDZ) treatment. This cross-sectional study revealed a specific frontal dysfunction in their unique sample and should reinforce the vulnerability of the prefrontal cortex due to sleep loss or disturbed sleep. This last assumption was further investigated by You et al. [4] analyzing the oxygen changes in the prefrontal cortex in short-sleep young adults with different physical activity levels during the execution of a Stroop task. The results showed not only the involvement of different brain regions in the cognitive performance but also the importance of a balance between physical activities and sleep for cognitive functioning in short-sleep population. Notably, D’Este et al. [5] evaluated the influence of cognitive reserve on cognitive performance in isolated rapid-eye-movement sleep behavior disorder (iRBD) patients. The results showed that, in this specific sleep disorder group, patients with high levels of cognitive reserve achieved the best performance in visuo-constructive and verbal memory functions and reported a lower percentage of mild cognitive impairment. Considering that the iRBD is recognized as the prodromal stage of neurodegenerative disorders, the protective role of the cognitive reserve for cognitive decline could explain the intersubject variability in the time of progression of this disease.

The importance of a good sleep quality for cognition was demonstrated in two studies in children with specific sleep disorders. In the first study, DelRosso et al. [6] assessed several cognitive abilities in a small sample of children with restless sleep disorder (RSD), abilities such as executive functions, attention, memory, cognitive processing, and language. This exploratory study revealed that children with RSD were mainly affected in selective attention, cognitive flexibility, and inhibitory control, in addition to daytime impairment, providing useful information for the clinicians. In the second study [7], children with different types (i.e., primary snoring, mild obstructive sleep apnea, and moderate/severe obstructive sleep apnea) of sleep-disordered breathing (SDB) were requested to perform an emotional expression recognition task. The authors found that children with moderate/severe obstructive sleep apnea tended to exhibit problems in facial expression categorization, with a possible impact in their social communication ability. The last approach presented in this Special Issue, as a tentative response to the scientific enigma of the function and regulation of sleep, assesses the changes in sleep quality in specific situations and context. On the one hand, the link between sleep and wake was studied through the relationship between habitual videogame playing and subjective sleep and daytime quality [8] and by the investigation of a specific phenomenon observed after morning awakening, that is, the sleep inertia [9]. In the first case, De Rosa et al. [8], through results of their online survey, revealed that participants who were habitual gamers showed delayed bed- and risetimes and were evening types, but they did not differ in daytime sleepiness and sleep quality with respect to nonhabitual gamers and non-gamer participants, suggesting a reconsideration of the impact of videogames on well-being. Using an ecological approach (i.e., motor activity measured using an actigraph) to evaluate the time course of sleep inertia dissipation in different age groups (from 9 to 70 years old), Tonetti et al. [9] report that sleep inertia was dissipated in 70 min after spontaneous morning awakening in line with the cortisol awakening response. In addition, the authors reported that the sleep inertia was more evident in younger people, suggesting again the importance of morning cortisol secretion for the decline in subjective sleepiness after the awakening. On the other hand, it is possible to investigate the sleep function and regulation in specific settings, such as prison [10]. Specifically, this particular environment plays an influence on sleep habits and sleep quality. This study [10] confirmed that prisoners showed more anxiety and depression than controls and that they reported a lower sleep quality with more signals of insomnia than the controls. Moreover, the objective measure of the sleep quantity indicated that prisoners reported longer sleep onset and less total sleep time.

This Special Issue provides a unique perspective on how sleep influences several cognitive functions and psychophysical well-being [11]. In addition, the importance of sleep can be seen throughout one’s life span, given that sleep is important for healthy growth and development [12]. Thus, the subjective and objective assessment of changes in sleep quality, as well as in daytime functioning, in several sleep disorders could not only shed light on the mechanisms of sleep function and regulation, but also provide useful information for clinicians in both diagnosis and treatment. This Special Issue could be considered a “starting point” along the way to elucidating the enigma of sleep.
